# Beyond the Terrestrial Narrative: Re-Evaluating the Ancient Coastal Lifestyle in Fuerteventura Through Fish Remains

**DOI:** 10.1007/s11457-026-09535-0

**Published:** 2026-07-31

**Authors:** Simon-Pierre Gilson, Aitor Brito-Mayor, Javier Adonay Cruz Viera, Rosa López Guerrero, Elías Sánchez-Cañadillas, Jonathan Santana

**Affiliations:** 1 Departamento de Ciencias Históricas, Universidad de Las Palmas de Gran Canaria, Las Palmas, Spain; 2 Grupo de Investigación Tarha, Departamento de Ciencias Históricas, Universidad de Las Palmas de Gran Canaria, Las Palmas, Spain; 3 Territorio y Sociedad, Evolución Histórica de un Espacio Tricontinental (África, América y Europa), Universidad de la Laguna and Universidad de las Palmas de Gran Canaria, La Laguna, Spain

**Keywords:** Fuerteventura, Ichthyoarchaeology, Majo, Fishing techniques and arts, Exploration of the territory

## Abstract

This study explores the relationship between the Majos of Fuerteventura, Canary Islands, Spain and the sea using ichthyoarchaeological analyses at Punta del Mallorquín, Llano del Sombrero, and Cueva de Villaverde. These sites provide data on fishing practices among the Majos. Combined with existing research, our results allow us to identify the taxonomic frequency of 27 species, offering an initial insight into the use of marine resources in indigenous times. Parrotfish (*Sparisoma cretense*) is the most frequent taxon identified. Based on available data, we propose both a specific fishing technique for parrotfish and a model of marine exploration linked to settlement patterns.

**Supplementary Information:**

The online version contains supplementary material available at https://doi.org/10.1007/s11457-026-09535-0.

## Introduction

The formation of the Canary Islands, which began between 35 and 30 million years ago, was driven by volcanic activity in the region (Anguita et al. 2025). Plants and animals from Africa gradually colonized these new territories (Fernández-Palacios and Whittaker 2008), but the earliest evidence of human presence dates from between the first century Before Current Era BCE and the second century Current Era CE, with Roman presence recorded on the Lobos islet. This site, dated to the first half of the second century CE, is interpreted as a seasonal workshop for purple dye linked to Roman activity in the Strait of Gibraltar (Del-Arco-Aguilar et al. 2016; Cebrián-Guimerá et al. 2022). However, the permanent colonization of the archipelago was carried out by Berber farming and fishing populations from North Africa between the first and third centuries CE (Serrano et al. 2023; Santana et al. 2024).

The islands of Lanzarote and Fuerteventura, along with their islets, present a highly eroded and arid landscape due to the limited influence of the trade winds and their proximity to the African continent. Their ancient inhabitants were known as Majos according to European written sources from the sixteenth and seventeenth centuries A.D. (Torriani 1590; Abreu Galindo 1632; Frutuoso 1964). These islands were sparsely populated, with genetic and archaeological evidence indicating episodes of significant demographic decline. There is still limited data available for Fuerteventura, which poses a challenge for researching demographic increases and declines on the island (Serrano et al. 2023; Santana et al. 2025). The material culture preserved in archaeological sites mainly consists of bone, mollusk, ceramic, and lithic industries, as the geology of the archipelago does not facilitate the presence of metallic minerals, nor have artifacts made from soft materials been preserved (Cabrera Pérez 1996; Lorenzo Perera 2016; Perera Betancor 2015; Lacave Hernández et al. 2024).

The presence of agricultural activity in Fuerteventura has been demonstrated through archaeobotanical identification of barley (*Hordeum vulgare*), wheat (*Triticum* cf. *durum*), and lentil (*Lens culinaris*) (Morales et al. 2023). In addition to goats, the study of faunal remains in Fuerteventura has identified the presence of sheep (*Ovis aries*), pig (*Sus domesticus*), domestic mouse (*Mus musculus*), and dog (*Canis familiaris*), as well as cat (*Felis catus*) on Lobos Island (Meco Cabrera et al. 1982; Carrascosa and López-Martínez 1988; Meco Cabrera 1992; Alcover et al. 2009; Siverio-Batista et al. 2021; González Quintero et al. 2025; Brito-Mayor et al*.*  2025). The evidence also suggests the utilization of wild resources, including marine species (monk seal, fish, and mollusks) and, to a lesser extent, native terrestrial flora and fauna (Meco Cabrera 1992; Rando and Perera 1994; Machado Yanes 1996; Morales et al. 2023; Del-Arco-Aguilar et al*.* 2025).

The exploitation of the sea by the first inhabitants of the Canary Islands is a scarcely researched topic from an ichthyoarchaeological perspective. Only five specific studies on the subject have been published, which adds to other works that mention fish remains, totaling more or less 9700 fish remains from 36 sites across various islands of the archipelago, and only one from Fuerteventura (Rodríguez Santana 1996; Rodrígues Santana 2002; Rodriguez Santana et al. 2008; Rodríguez Rodríguez et al. 2021; Cabrera Sosa et al. 2022). These studies, with the most important being Carmen Gloria Rodríguez Santana’s doctoral thesis (1996), provide information about the islands of Gran Canaria, La Palma, Tenerife, La Gomera, and El Hierro. For Fuerteventura, the presence of fish remains is mentioned without quantitative data at the Tindaya site, with the identification of parrotfish (*Sparisoma cretense,* Linnaeus, 1758) (Velasco-Vázquez et al. 2000), and at the Cueva del Junquillo with remains of parrotfish, salema (*Sarpa salpa*, Linnaeus, 1758), comber fishes (*Serranus* spp.), moray eels (Muraenidae), and seabreams (*Diplodus* spp.) (Suleiman Ruiz and López Guerrero 2025). Additionally, the presence of unidentified fish remains is reported at the Cueva de los Ídolos (Castro Alfín 1975), Butihondo (Del Arco-Aguilar et al*.* 2006), Valle de la Cueva (González Quintero et al. 2025), and on Lobos Island (Siverio-Batista et al. 2021).

Stable isotope studies conducted in the Canary Islands have allowed us to study the diet of the indigenous inhabitants (Tieszen et al. 1995; Arnay-de-la-Rosa et al. 2010; Lécuyer et al. 2021; Sánchez-Cañadillas et al. 2021, 2023, 2026). These initial studies suggest limited consumption of marine products in the diet, except in El Hierro, where significant intake of low-trophic-level marine elements, interpreted as mollusk fauna, has been observed (Arnay-de-la-Rosa et al. 2010). A greater role for these foods is also noted in the final moments of pre-Hispanic occupation in Gran Canaria, coinciding with the onset of the Little Ice Age (Lécuyer et al. 2021). However, stable isotope studies of insular populations tend to underrepresent marine resources because the terrestrial protein signal often overlaps with the marine signal (Kinaston et al. 2013). Additionally, due to overexploitation and extensive construction in coastal areas, few archaeological sites are preserved along the islands’ coastlines, so most human remains studied in the archipelago come from inland mid-elevation zones. Therefore, except at some sites (primarily Gran Canaria and El Hierro), paleodietary information is available only for populations that lived and conducted burial activities inland. Similarly, no previous studies on stable isotopes in marine remains from archaeological contexts have been conducted, so the exact values of the marine food sources in the archipelago during the pre-Hispanic period are unknown (Sánchez Cañadillas et al*.* 2026).

Island archaeology has shown that insular subsistence systems emerge from the interaction between land and sea under the constraints of insularity (size, isolation, seasonality, and marine potential), so mixed economies are often the norm rather than the exception (Kirch 2000; Erlandson and Fitzpatrick 2006; Rainbird 2007). In this context, ichthyoarchaeology provides an empirical contrast of subsistence strategies by allowing the inference of capture areas, technology, logistics, and processing and consumption practices, which contribute to revising traditional interpretive narratives that have undervalued marine contributions (Erlandson 2001; Erlandson and Rick 2010; Fitzpatrick and Erlandson 2018). In the Canary Islands, where archaeological literature has tended to project a pastoral bias when considering the economic strategies of the Canary islanders, especially in the case of Fuerteventura (Meco Cabrera 1992; Cabrera Pérez 1996), fish assemblages allow for quantifying the coastal contribution, refining secular interpretations of the pre-European past of the islands.

In this context, the present work aims to examine the relationship between the Majos of Fuerteventura and the sea through ichthyoarchaeological analysis of the sites at Punta del Mallorquín, Llano del Sombrero, and the Cave of Villaverde. Here, the data obtained on the Majos’ fishing activity are presented. This exploitation appears to reflect a utilization of the maritime resources linked to a dynamic occupation of the territory.

### Archaeological Context of the Ichthyological Archaeological 

**Fig. 1:**
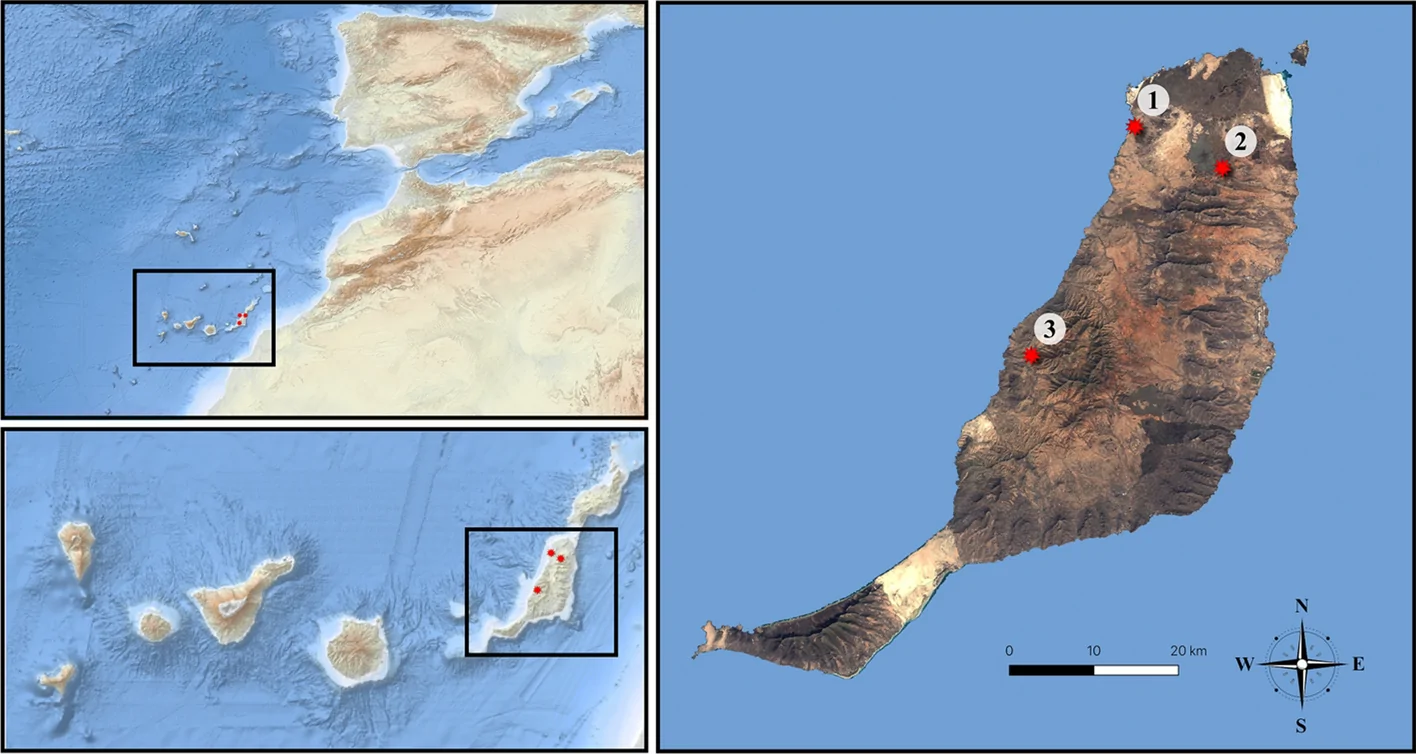
Geographic location of the sites: (1) Punta del Mallorquín, (2) Villaverde Cave, (3) Llano del Sombrero

Three faunal assemblages from three key archaeological sites in Fuerteventura were analyzed: Punta del Mallorquín, Llano del Sombrero, and the Cave of Villaverde (Fig. 1). The general fauna assemblage derives from recent excavation campaigns (Brito-Mayor et al., under review). Additionally, the ichthyological dataset integrates remains recovered during earlier fieldwork that had remained unpublished, thereby expanding the taxonomic and size representation of the fish record at each site.

Punta del Mallorquín is defined as a domestic and food processing site, characterized by the presence of a shell midden. Excavated between 2017 and 2018, it has been dated to the fifth and sixth centuries A.D. (López Guerrero and Castellano Fernández 2019).

Llano del Sombrero is an open-air site with domestic structures, interpreted as a residential space (Castañeyra Ruiz and López Guerrero 2014). Its occupation spans from the 8th to the sixteenth century and is divided into three phases: Phase I (eighth–ninth centuries): corresponds to an accumulation of food debris. Phase II (eleventh–twelfth centuries): interpreted as the interior part of a construction. Phase III (sixteenth century): characterized by a fill with burned material and imported ceramics after European contact (López Guerrero 2017). The fish remains studied come exclusively from Phase I (eighth–ninth centuries).

Villaverde Cave is a volcanic tube that was initially used as a dwelling space and later as a funerary zone. Its use spans from the third to the twelfth century AD (Hernández and Sánchez 1990; López Guerrero 2025, 2019, 2021; López Guerrero et al. 2020) and can be divided into four phases. Phase I (third–fifth centuries AD): documented in the deepest stratigraphic layers inside the cave. Phase II (sixth–eighth centuries AD): characterized by continued occupation inside the volcanic tube and an increase in construction activity on the exterior of the cave. Phase III (ninth–tenth centuries AD): associated with intensive use of the structures inside the cave. Phase IV (eleventh–twelfth centuries AD): marks the abandonment of the cave as a dwelling space and its reuse as a funerary zone.

## Methodology

### Sampling Methodology

The methodology applied for collecting the faunal material in the excavations at Punta del Mallorquín and Cueva de Villaverde consisted of dry screening with a 3 mm (mm) mesh for the fill levels and flotation for the levels associated with occupation episodes using a 1 mm mesh (López Guerrero 2017, 2019; López Guerrero and Castellano Fernández 2019; López Guerrero et al. 2020). This sampling methodology allowed for the recovery of the greatest possible amount of potentially preserved material remains at the archaeological site, especially smaller animals such as fish. At the Llano del Sombrero site, the method involved only manual collection, which introduces a significant bias in terms of collected material when compared to the other two sites analyzed.

Applying two different sampling methodologies involves a series of limitations in the study of the faunal material. First, different collection strategies generate a bias toward animals of different sizes (Chaix and Méniel 2001; Reitz and Wing 2008; Gifford-Gonzalez 2018). Second, the sorting of floated sediments by non-specialized personnel in animal remains can increase the loss of important elements, such as fish otoliths, a common scenario in the case of the Canary Islands.

### Methodology for the Study of the Faunal Material

The faunal material sampled during the excavation campaign was studied with the support of the faunal reference collection of the Archaeology Laboratory at ULPGC (Las Palmas Zooarchaeology, LPZ-ULPGC). Additionally, the collection from the Museum and Archaeological Park Cueva Pintada and available reference works (Olsen 1968) were used. The number of specimens analyzed (NSA) was divided into two groups: Number of identified specimens (NISP), which prioritizes the most specific taxonomic category; and number of unidentified remains (UNI), those that could not be taxonomically classified (Reitz and Wing 2008). All faunal remains were quantified using the Minimum Number of Individuals (MNI), considering the size of the elements as an indicator of different individuals (Lyman 2008).

The fish remains were measured following the standardized procedures by Morales and Rosenlund (1979) and Rodríguez Santana for the parrotfish (1996).

The estimation of the size and weight of the parrotfish (*Sparisoma cretense*) was based on regression calculations published by Rodríguez Santana (1996) and Jurado-Ruzafa and Martín-Sosa (2022). For other species, due to the lack of available regression calculations, size was estimated by comparing with reference collections from the Archaeology Laboratory at the University of Las Palmas de Gran Canaria (Las Palmas Zooarchaeology) and the collection of the Cave Pintada Archaeological Park and Museum. The weight of these individuals was estimated using regression calculations available in the literature for each species (Torres 1991; Crawford 1993; Pajuelo and Lorenzo 1995; Gonçalves et al. 1997; Andrade et al. 2003; IGFA 2009; Oliveira et al. 2015; Rodríguez-García et al. 2023). For the shark, the animal’s size was estimated using the regression equation published by Casey et al. (1985), and the weight was determined using the method proposed by Basusta (2016). It is worth noting that all the measurements taken came from bones and not otoliths (see supplementary), which are rarely found at sites in the Canary Islands.

The calculation of the meat value of mammals presented here is based on MNI values, estimated age categories obtained by Brito-Mayor and co-authors (under review), and the known weight for current goat livestock in the Canary Islands (López Fernández et al. 1993; Salvador et al. 2009) as well as the observed weight for pigs in ethnographic studies (Halstead and Isaakidou 2011; Lorenzo Perera 2016).

Thermal alterations were recorded following the methodology proposed by Costamagno and co-authors (2009), as well as the other taphonomic variables potentially reflected in the ichthyoarchaeological material (Frontini et al. 2022; Zohar et al*.* 2025).

The statistical tests (*χ*^2^, Fisher’s test, ratification curve) were performed using the Past software (v4.15), and the descriptive statistics (scatter plot, box plot, histogram) were conducted with IBM Statistical Package for the Social Sciences (SPSS software) (v25).

## Results

### General Description of the Sample

The ichthyofaunal collection from the three sites is composed of 1554 bone fragments (Table 1). The analysis allowed for the identification of 84% (Number of Identified Specimens [NISP] = 1303) of the remains at the genus and species level. It was not possible to identify 16% (NISP = 251) of the fragments beyond the infraclass Teleostei.

**Fig. 2:**
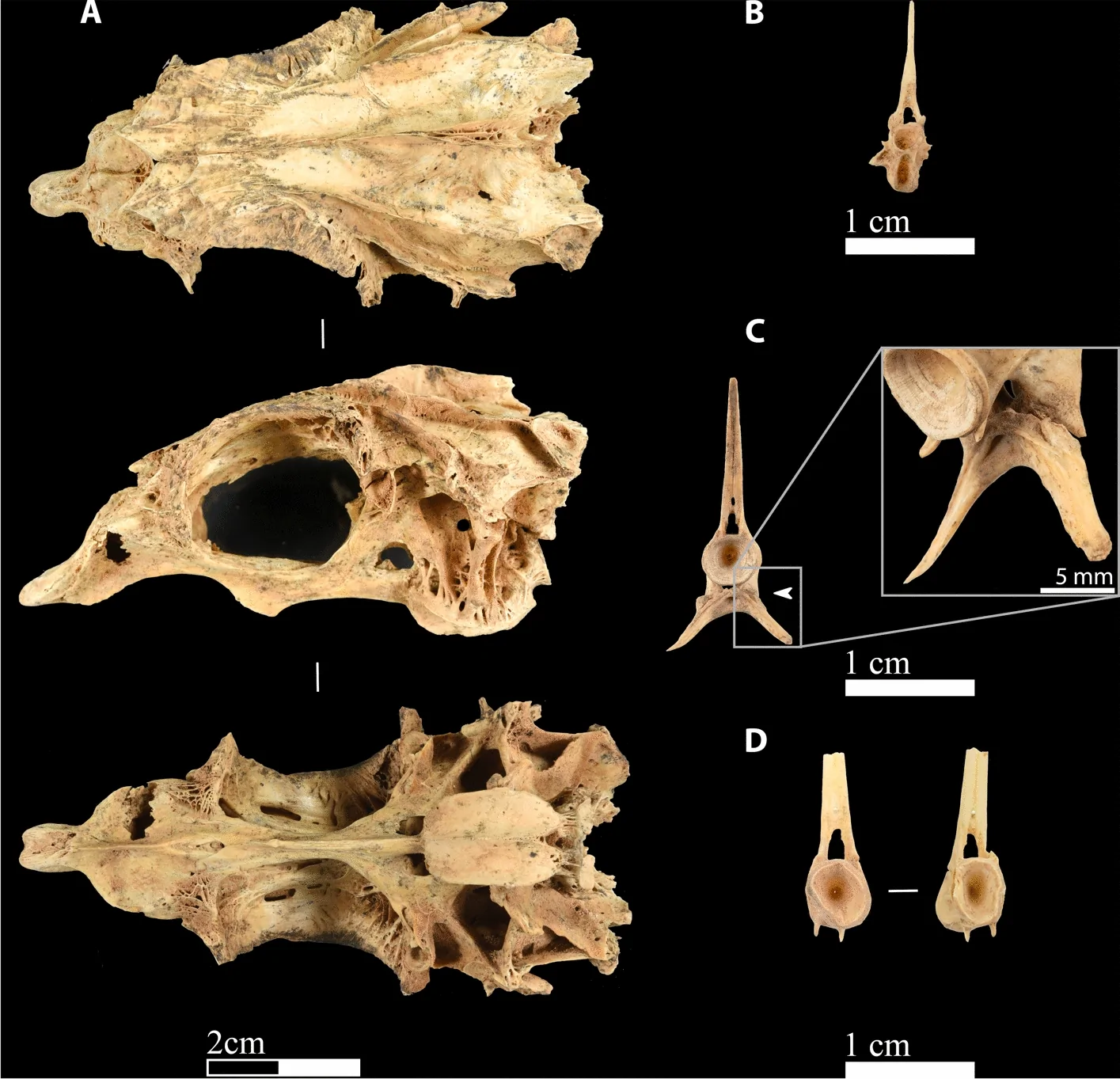
**A** Complete neocranium of a parrotfish (*S. cretense*) from the Villaverde Cave site; **B** Deformed vertebra of a bogue (*B. boops*) caused by compression; **C** Vertebra of a parrotfish (*S. cretense*) with a possible cut mark; **D** Vertebra of a parrotfish (*S. cretense*) deformed by compression

The remains are in good condition, with relatively low fragmentation, especially in the vertebrae, where it is mainly limited to loss of the spinous processes. In the Villaverde Cave, the preservation is particularly remarkable, with the presence of a complete parrotfish neurocranium (Fig. 2) and four nearly complete ones.

### Taxonomic Identification

The study of the ichthyological remains from the three sites has revealed the presence of 30 taxa: 4 at genus level and 26 at species level, with some specimens only identified at family level (Table 1).

**Table 1:** Taxonomic identification of the fish remains found at the sites of Cueva de Villaverde, Punta del Mallorquín, and Llano del Sombrero

Species	Cueva de Villaverde (3th–12th CE)				Punta del Mallorquin (5th–6th CE)				Llano del Sombrero (11th–13th CE)			
	NISP	%NISP	MNI	%MNI	NISP	%NISP	MNI	%MNI	NISP	%NISP	MNI	%MNI
Elasmobranchii												
* Carcharhinus* sp.	1	< 1	1	1								
Teleostei	96				160							
Aulostomidae												
* Aulostomus* *strigosus*					1	< 1	1	3				
Berycidae												
* Beryx splendens*					1	< 1	1	3				
Carangidae												
* Pseudocaranx dentex*					2	1	1	3				
* Seriola fasciata*					1	< 1	1	3				
* Trachinotus ovatus*	7	< 1	3	3								
Muraenidae												
Muraenidae	1	< 1	1	1	28	13	1	3				
* Muraena augusti*					2	1	2	5				
* Muraena helena*					1	< 1	1	3				
Epinephelidae	1	< 1			1	< 1						
* Epinephelus marginatus*	1	< 1	1	1	2	1	1	3	1	14	1	50
* Mycteroperca fusca*	10	1	5	5								
Haemulidae												
* Parapristipoma octolineatum*					1	< 1	1	3				
* Pomadasys incisus*	1	< 1	1	1								
Pomacentridae												
* Chromis limbata*	1	< 1	1	1								
* Similiparma lurida*					1	< 1	1	3				
Sparidae	3				11	5						
* Boops boops*	3	< 1	1	1								
* Dentex cf. gibbosus*	1	< 1	1	1								
* Diplodus* spp.					2	1	1	3				
* Diplodus annularis*	3	< 1	3	3	2	1	2	5				
* Diplodus cadenati*	5	< 1	2	2								
* Diplodus cervinus*	4	< 1	2	2								
* Diplodus* cf.* puntazzo*	1	< 1	1	1								
* Oblada melanura*					1	< 1	1	3				
* Pagellus erythrinus*	1	< 1	1	1								
* Pagrus* sp.	1	< 1	1	1								
* Pagrus auriga*	5	< 1	2	2	1	< 1	1	3				
* Pagrus pagrus*	1	< 1	1	1	2	1	1	3				
* Sarpa salpa*	22	2	6	6	6	3	2	5				
* Spondyliosoma cantharus*					1	< 1	1	3				
Labridae Scarinae												
* Sparisoma cretense*	1003	93	73	68	153	70	17	46	6	86	1	50
Total	1172	100	107	100	380	100	37	100	7	100	2	100

In the table, cf. stands for *confer*, when placed between a genus and species name, it indicates the identifier is uncertain but strongly suspects the specimen belongs to that specific species or a very closely related one; sp. is used when a specimen is identified at the genus level, but the specific species is either unknown or unidentifiable; and spp. is used when referring to several unidentified specimens in a same genus but without specific identification to none

The parrotfish *(S. cretense*) is the most common species across the three sites, both in terms of the number of identified remains (NISP = 1162; % NISP = 90%) and the minimum number of individuals (MNI = 91; % MNI = 62).

The presence of moray eels (NISP = 32) has also been confirmed, including the black moray (*Muraena augusti*, Kaup, 1856) and the painted moray (*Muraena helena*, Linnaeus, 1758).

The Epinephelidae are also present (NISP = 16), represented by the island grouper (*Mycteroperca fusca*, Lowe, 1838) and the dusky grouper (*Epinephelus marginatus*, Lowe, 1834).

The Sparidae represent another important part of the identified species, highlighting the salema (*S. salpa*) and various species of seabream: *Diplodus annularis* (Linnaeus, 1758)*, Diplodus cadenati* (de la Paz, Bauchot & Daget, 1974)*, Diplodus cervinus* (Lowe, 1838)*,* and *Diplodus puntazzo* (Walbaum, 1792).

### Anatomical Identification

The set of fish bone remains mainly consists of vertebrae (57%, 939/657), followed by bones of the viscerocranium (25%, 421/1657), the neurocranium (2%, 40/1657), the pectoral girdle (1%, 21/1657), and the pelvic girdle (< 1%, 3/1657), with 5% of indeterminate bones (Table 2).

**Table 2:** Anatomical identification of fish remains found at the sites of Cueva de Villaverde, Punta del Mallorquín, and Llano del Sombrero

Category	Cueva Villaverde		Punta del Mallorquín		Llano del Sombrero	
	NISP	%NISP	NISP	%NISP	NISP	%NISP
Axial skeleton	840	67	236	62	5	71
Vertebrae	5	0,4	15	4		
Atlas	21	2	3	1		
Vertebrae thoracalis	70	6	16	4	1	14
Vertebrae praecaudalis	143	11	15	4	1	14
Vertebrae caudalis	524	41	101	27	3	43
Urostylus	17	1	4	1		
Spina	21	2	41	11		
Processus spinosus	39	3	41	11		
Neurocranium	25	2	2	1		
Basioccipitale	8	0,6				
Exoccipitale	1	0,1				
Frontale	9	1				
Opisthoticum			1	0,3		
Parasphenoideum	4	0,3	1	0,3		
Sphenoticum	2	0,2				
Vomer	1	0,1				
Viscerocranium	339	27	80	21	2	28
Pharyngeum superius	100	8	19	5	2	28
Pharyngeum inferius	70	6	13	3		
Epibranchiale	4	0,3	2	1		
Epihyale	1	0,1				
Hypobranchiale			1	0,3		
Keratobranchiale			1	0,3		
Dentale	48	4	1	0,3		
Articulare	8	0,6	1	0,3		
Praemaxilare	60	5	12	3		
Maxilare	6	0,5	4	1		
Tooth	3	0,2	10	3		
Palatinum	4	0,3	4	1		
Mesopterygoideum	1	0,1				
Metapterygoideum	2	0,2	1	0,3		
Lacrimale	1	0,1				
Quadratum	10	1	2	1		
Praeoperculare	4	0,3	3	1		
Pterygoid	1	0,1				
Operculare	7	0,6	3	1		
Interperculare			2	1		
Hyomandibulare	9	0,7	1	0,3		
Cintura escapular	17	1	4	1		
Cleithrum	5	0,4	1	0,3		
Coracoideum	1	0,1				
Postcleithrale	1	0,1				
Scapula			2	1		
Supracleithrale	10	1	1	0,3		
Cintura pélvica	2	0,2	1	0,3		
Basipterygium	2	0,2	1	0,3		
Indeterminated	27	2	57	15		
Total	1250	99	380	100	14	100

### Bone Alterations

A vertebra of parrotfish (*S. cretense*) and another from an unidentified fish show signs of compression at the Punta del Mallorquín site. In Phase II of the Villaverde Cave (sixth–eighth centuries CE), signs of compression have been documented on a salema (*S. salpa*), a dusky grouper (*E. marginatus*), and two bogue (*Boops boops*, Linnaeus, 1758) vertebrae.

Two cut marks with a lithic instrument were recorded, both on parrotfish vertebrae: one in the Villaverde Cave and the other at Punta del Mallorquín.

The action of fire has been evidenced in 61 remains from Punta del Mallorquín and in 381 remains from the Villaverde Cave. For Punta de Mallorquín, charred bones represent 23% of the total burned bones, and only 5% in the case of Villaverde Cave. In Villaverde Cave, there is one calcined bone, compared to zero in Punta de Mallorquín.

### Osteometry

Osteometric data of the parrotfish (*S. cretense*) have been collected from the three archaeological sites, totaling 331 bones measured. Considering the stratigraphic, spatial, and anatomical data, to avoid including bones from the same fish in the calculations, 83 parrotfish fish bones were selected: 67 from the Villaverde Cave (Phase I: 13, Phase II: 7; Phase III: 43, Phase IV: 4), 15 from Punta del Mallorquín, and 1 from Llano del Sombrero. The selection of bones followed the same rule as for the NMI calculation, taking into account the stratigraphy, the most frequently found bone, and the estimated size.

Considering the dataset, the average estimated size of the parrotfish is 32 cm (cm), with an average estimated weight of 564 g (g). The largest specimen reaches 46 cm and weighs 1509 g, while the smallest has 22 cm and weighs 168 g. The standard deviation of the size is only 5 cm, with a low coefficient of variation of 15%. These numbers indicate a high level of stability among the deposits analyzed over time (Table 3).

**Table 3:** Mean, median, standard deviation, and coefficients of variation of the total length calculated in centimeters of the parrotfish (*Sparisoma cretense*)

	Mean (cm)	Median (cm)	Standard deviation	CV
Villaverde				
Phase I (3th-5th CE)	33	32	4.4	0.13
Phase II (6th-8th CE)	32	31	5.5	0.17
Phase III (9th-10th CE)	32	32	4.8	0.15
Phase IV (11th-12th CE)	34	34	5.8	0.17
Punta Mallorquin				
Phase I and II (5th-6th CE)	32	32	5	0.16
All sites and phases	32	32	5	0.15

**Fig. 3:**
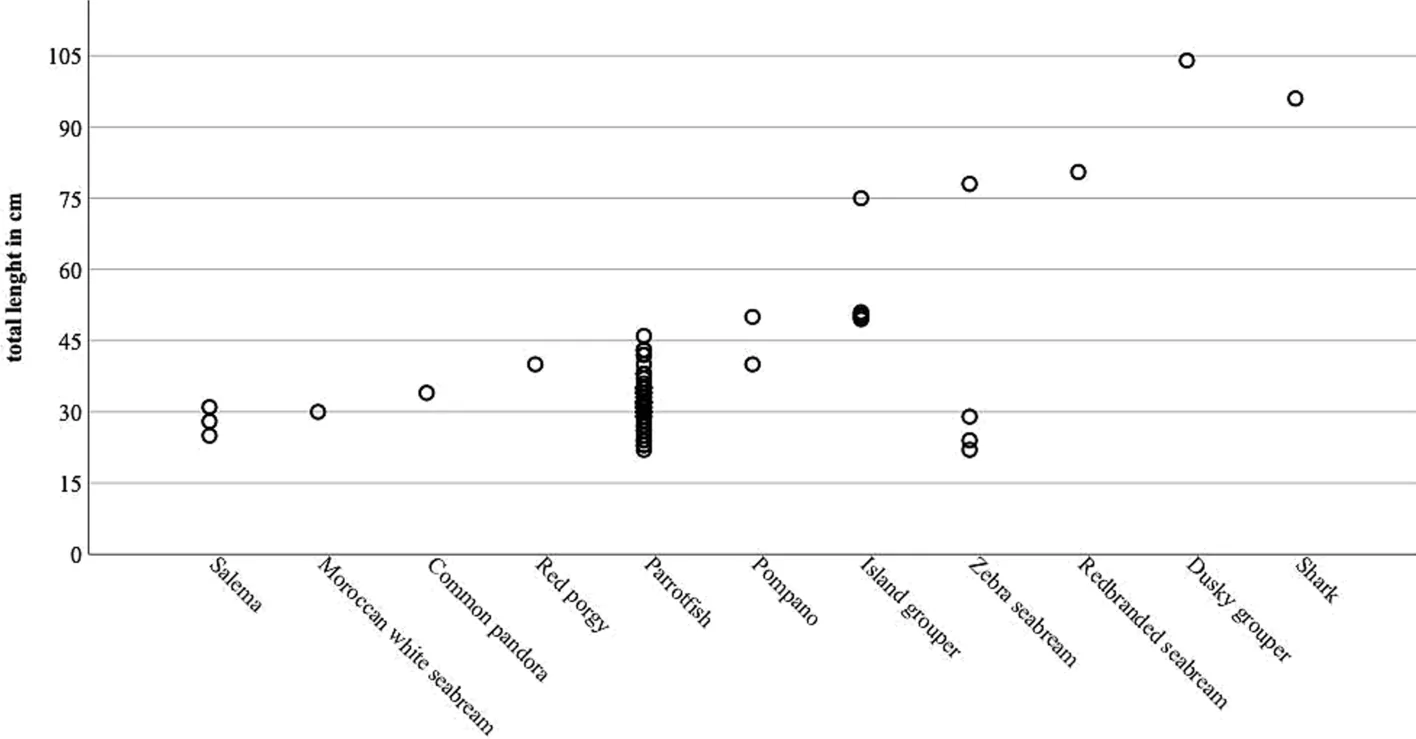
Diagram of the estimated total length in centimeters of the fish in the Villaverde Cave: Salema (*Sarpa salpa*), Moroccan white seabream (*Diplodus cadenati*), Common pandora (*Pagellus erythrinus*, Linnaeus, 1758), Red porgy (*Pagrus pagrus*, Linnaeus, 1758), Parrotfish (*Sparisoma cretense*), Pompano (*Trachinotus ovatus*, Linnaeus, 1758), Island grouper (*Mycteroperca fusca*), Zebra seabream (*Diplodus cervinus*), Redbanded seabream (*Pagrus auriga*), Dusky grouper (*Epinephelus marginatus*), Shark (*Carcharinhus* sp.)

The estimation of the size and weight of eleven other species identified in the Villaverde Cave has also been carried out, showing the existence of large animals, such as a dusky grouper (*E. marginatus*) measuring 104 cm and weighing 17.5 kg [kg], a redbanded seabream (*Pagrus auriga*, Valenciennes, 1843) measuring 80 cm and weighing 9.9 kg, and a zebra seabream (*D. cervinus*) measuring 78 cm and weighing 6.9 kg (Fig. 3).

## Discussion

This ichthyoarchaeological analysis offers initial insight into fish exploitation by the Majo people of Fuerteventura, and the interpretations should be treated only as initial hypotheses to be tested in future analyses. Recovery methods varied across sites and significantly shaped the samples. Punta del Mallorquín and Cueva de Villaverde, where fine sieving was used, contain a large number of fish remains (99.5% NISP). In contrast, Llano del Sombrero, excavated without sieving, shows very few fish remains (0.5% NISP). This difference demonstrates that the absence of fine-mesh recovery leads to underrepresentation of small-bodied taxa, thereby underestimating the importance of fish in Majo subsistence.

The faunal collections studied, originating from the three sites, present 30 fish taxa. The habitats of these species, at both the family and specific taxon levels, are mostly associated with rocky bottoms and shallow depths. However, some specimens are also recorded in intertidal pools, such as the Seabream (*Diplodus* spp.). Additionally, species characteristic of deeper waters have been identified, including the amberjack (*Seriola fasciata*, Bloch, 1793) and the shark (*Carcharhinus* sp.). Regarding the latter, it is worth noting that various species of this family are known to approach coastal areas, a circumstance favored in the Canary Islands, where the coastal bathymetry drops sharply, facilitating the presence of pelagic fauna in coastal waters (Brito et al. 2002; Voigt and Weber 2011; Ebert, Fowler, and Compagno 2016; Varela et al. 2025).

**Fig. 4:**
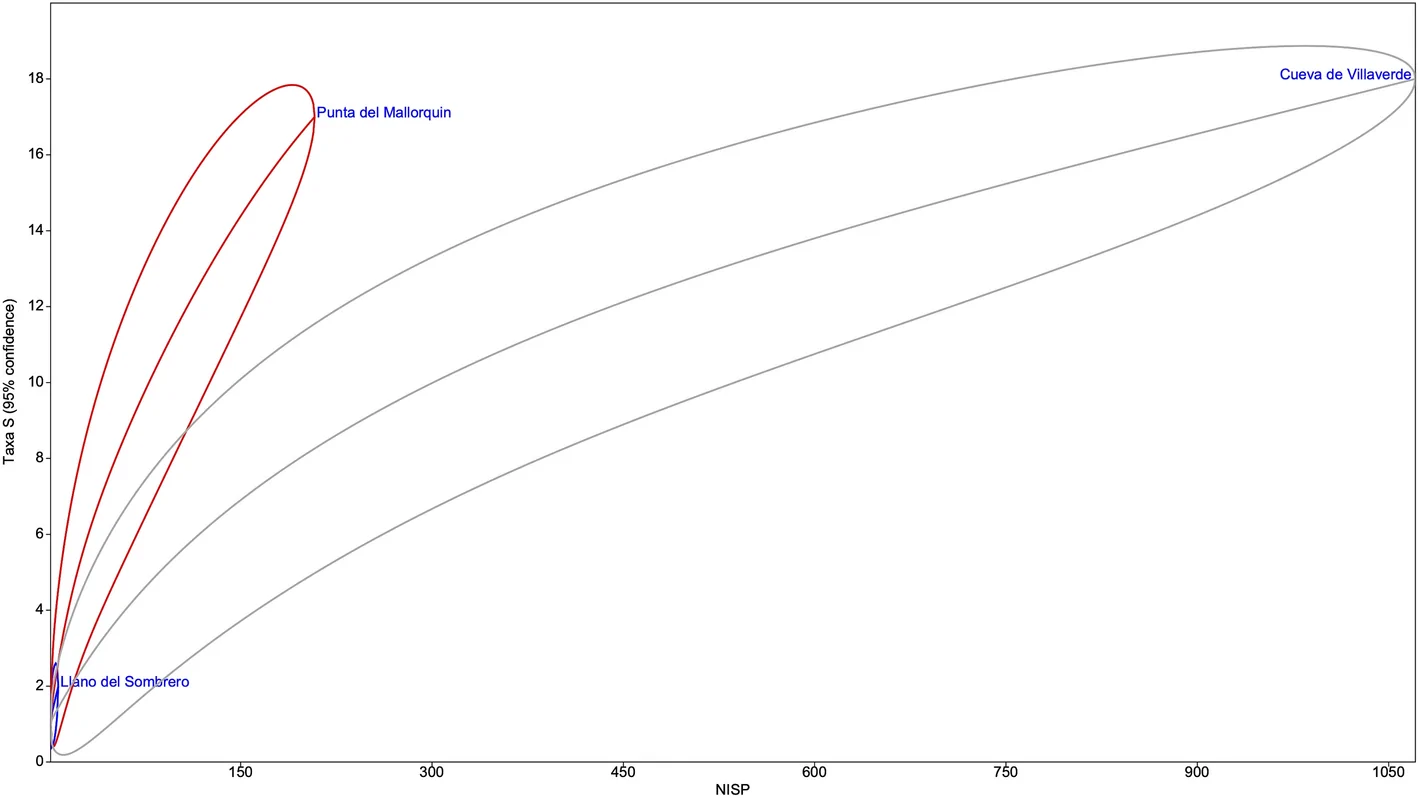
Rarefaction curve of the 3 sites with a 95% confidence interval

In terms of diversity, 17 different species have been identified at both the Punta del Mallorquín site and the Villaverde Cave. However, as shown by the rarefaction curve (Fig. 4), species diversity is higher at Punta del Mallorquín, considering that this site has a greater number of species relative to the total number of identified remains (Fig. 4). This is consistent with the interpretation of Punta del Mallorquín as a site where activities related to processing marine products took place, whereas the Villaverde Cave was a habitation space where only a portion of the collected items was brought. The greater diversity at Punta del Mallorquín is even more pronounced when considering the accumulation time. In fact, all the bones studied from Punta del Mallorquín come from a stratigraphic record with a maximum time interval of 200 years (5th–6th centuries), in contrast to the chronological span of Villaverde, which extends over 900 years (3rd–12th centuries). This diversity of species, both within and across sites, indicates active exploitation of different coastal zones. It is interesting to note that, in both cases, as evidenced by the absence of a plateau in the curves (Fig. 4), the total diversity of exploited fish was not recorded at either site, suggesting an even greater diversity of utilization than has been documented so far.

The parrotfish (*S. cretense*) has the highest number of remains, both in NISP (90%) and MNI (62%). The dominance of the parrotfish is a common phenomenon within the ichthyoarchaeological collection studied in the Canary Islands (Rodríguez Santana 1996, 2002; Rodríguez Rodríguez et al. 2021; Cabrera Sosa et al. 2022) and in all sites with fish records in Fuerteventura (Velasco-Vázquez et al. 2000; Suleiman Ruiz and López Guerrero 2025). However, it is important to remember that this is not always the case, such as the massive presence of sardines (Alosidae, previously Clupeidae) at the Cueva pintada (Rodríguez Santana 1996) and at Lomo de los Melones (Rodríguez Santana et al. 2008), as well as the garfish (*Belone belone*, Linnaeus, 1760) at Playa Chica (Santana et al. 2026).

Regarding the importance of the parrotfish within Canarian indigenous society, the weight calculation performed at the Villaverde Cave provides interesting information. In fact, in terms of meat, the parrotfish fish contributes 38 kg (36%), while other fish account for 68 kg (64%), contrasting with the number of identified remains (parrotfish: 90% NISP) and the minimum number of individuals (parrotfish: 62% NMI). This difference is mainly due to the size attained by other species, such as the dusky grouper (*E. marginatus*) (104 cm) or the island grouper (*M. fusca*) (50–75 cm). Thus, a single fish of these species can provide a greater amount of meat than several parrotfish. Therefore, it is important to note that, although the parrotfish may be the most frequent in terms of remains and possibly in terms of catch, its contribution to the diet is not as dominant as the counts might suggest. This highlights the importance of contextualizing quantitative data in zooarchaeological analysis. The preservation and identification of a bone (or fragment) are subject to numerous variables related to the bone’s own structure and its relationship with taphonomic processes. In this regard, the bones of the parrotfish show greater resistance than those of many other fish species, especially pharyngeal bones. This favors their greater representation in archaeological collections, as demonstrated by the percentage of skeletal part representation (%SPR, see supplementary material). The %PR of the vertebrae of the parrotfish in Villarverde Cave (35%) and Punta del Mallorquín (23%) is much higher than in other species (11% and 12% for the bream; 1% and 5% for the morays). The pharyngeal bones are represented at 60% and 70%.

In the same vein, mollusk remains are much better preserved than bones and create a visual effect that influences our perception, and consequently, their importance within the diet could be overestimated. However, many studies (for example, Figuti 1993) have shown that even with an extremely higher number of shells compared to bones, the amount of meat produced by those animals is much less than that of fish. In fact, the biomass in mollusks is usually less than 30% of the total mass of the animal, while in vertebrates, biomass is typically over 70% (Reitz and Wing 2008).

In terms of meat quantity, the Villaverde Cave and Punta del Mallorquín offer case studies to compare the value of fish within the diet of the Majos. Thanks to the study conducted on mammal remains by Brito-Mayor and co-authors (under review), it has been possible to calculate the amount of meat from the main species of the Majo livestock (goat, sheep, and pig) for both sites. This initial comparison is still exploratory but allows, for the first time, this approach to be made in two Canary Islands archaeological sites. It should also be remembered that the meat value does not represent the total meat obtained by the site’s inhabitants but rather a comparative value that allows for assessing the relative contribution of each resource source. The result of this calculation shows that at Villaverde Cave, livestock accounts for 95% (1548 kg) compared to 5% (84 kg) for fish (see supplementary). This value is slightly lower than the average 10% estimated through stable isotopes for the entire islands (Santana et al. 2024). This lower value was expected, as Villaverde Cave is a domestic site located away from the coast where only some fish bones were transported, thus minimizing their participation in the diet. In fact, at the Punta del Mallorquín site, the contribution of fish reaches 20% (see supplementary), and the total marine contribution would be even higher if the biomass produced by marine invertebrates, such as limpets, crabs, and sea urchins, is included.

**Fig. 5:**
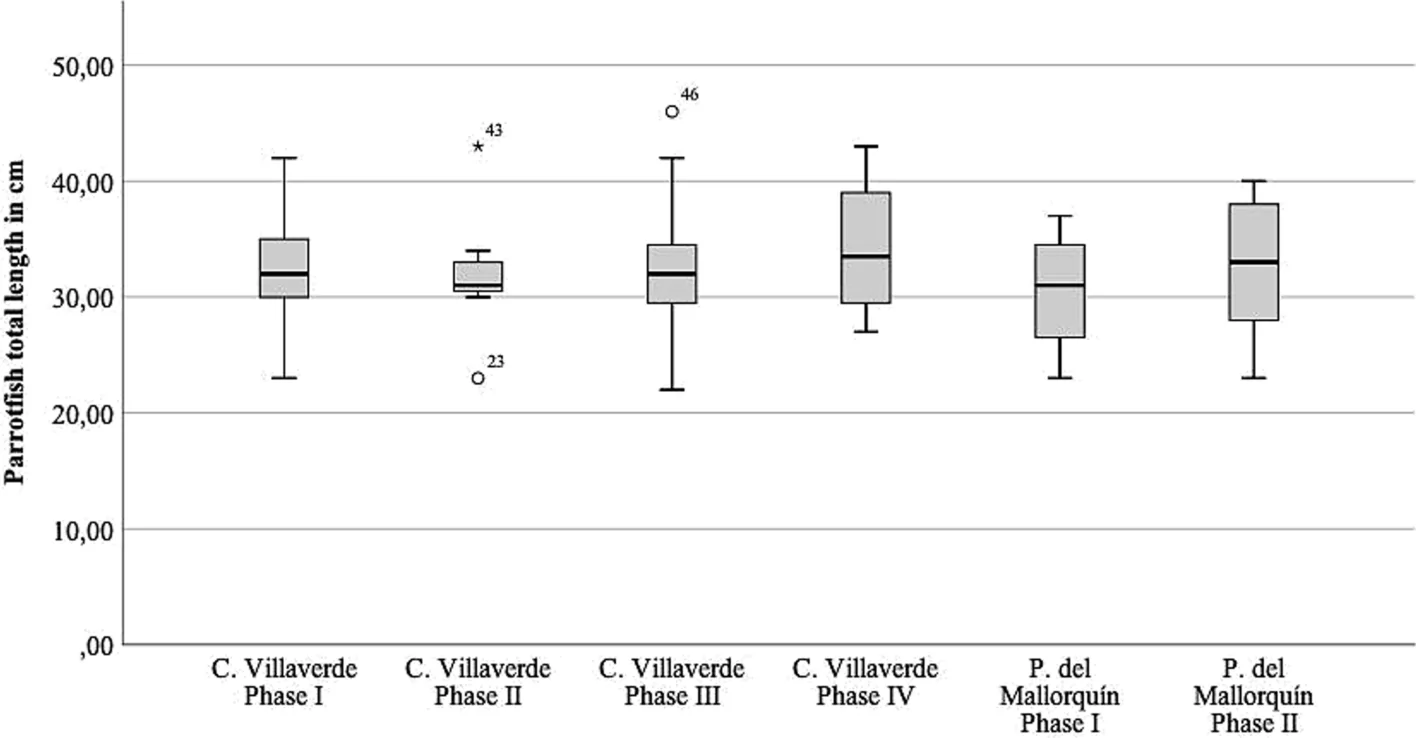
Box plot of the estimated total length of the parrotfish (*Sparisoma cretense*) in the different phases of the Villaverde Cave and Punta del Mallorquín

The osteometric data collected for the parrotfish also suggest the existence of a controlled fishing technique, aimed at individuals larger than 20 cm with an average capture size between 30 and 35 cm This technique has been present since the beginning of human presence in Fuerteventura, as shown by the data from Phase I of the Villaverde Cave and it persists throughout the entire indigenous period, as supported by the data collected in all phases across the three sites (Table 3, Fig. 5). In fact, both the mean and median remain very similar across the three sites and throughout all phases (Table 3, Fig. 5). These values are very similar to the osteometric data obtained by Rodríguez Santana (1996, 2002) for the other islands in the archipelago. Considering that the length estimations are based on several different bones rather than on otoliths (see supplementary), and given the one mm sieve used at Villaverde and Punta del Mallorquín, the osteometric dataset for the parrotfish seems to offer a reliable picture of the size of the animals caught.

Fish can be caught using a wide variety of techniques, ranging from the use of fishing handlines to the use of birds (Reitz and Wing 2008). Identifying techniques and methods from archaeological records is not straightforward and requires consideration of both zooarchaeological data and the biological behavior of the fish and its responses to fishing strategies (Reitz and Wing 2008). When available, written and ethnological sources are also valuable. For the Canary Islands, narrative sources mention the presence of nets, hooks, traps (or at least a trapping system), and the use of artificial intertidal pools associated with poison fish technique (Rodríguez Santana 1996). For the island of Fuerteventura, according to the documentary made by Monesma (2021), an old traditional fishing method still exists, as is also the case in Gran Canaria (Suárez Moreno 2004). This technique is characterized by the use of a bare shrub branch rod, lacking a reel, at the distal end of which is attached an extension made from horn covers from goats, carefully polished and assembled. This end piece increases the instrument’s sensitivity when detecting the bite of the parrotfish at the end of the fishing line.

**Fig. 6:**
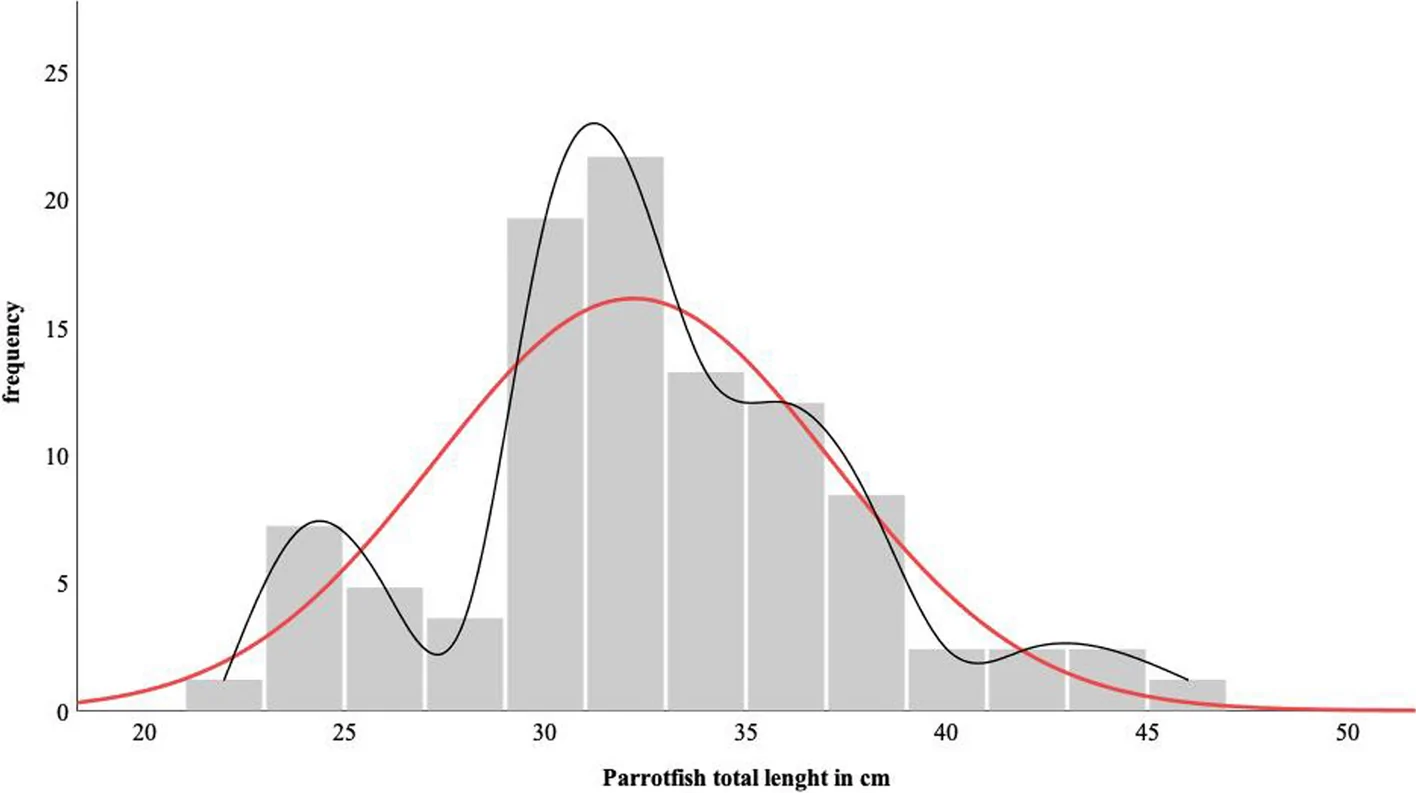
Bar graphic representation of the Parrotfish (*Sparisoma cretense*) comparing the estimated total length of the fish and their percentage frequency across the three sites and all phases. The normal distribution curve (in red) is also shown

Thus, for the parrotfish, considering its habits, the selection index (= 1.6 based on current data of parrotfish fish from Fuerteventura published by Falcón and co-authors in 1996) and the projection of the mortality profile (Fig. 6), which shows the presence of a selection of adult animals (over 20 cm) for the set of sites studied here, as well as the presence of a still-living traditional fishing art, it is possible to hypothesize a similar practice by the indigenous groups of Fuerteventura. Therefore, it is reasonable to consider that the capture of the parrotfish was most likely carried out using hook-and-line fishing gear, which may or may not have been associated with the use of a fishing rod. In fact, another hook-and-line fishing technique, without a rod, is known in the Canary Islands as the ‘puyón’ technique. This fishing method is currently specific to parrotfish and practiced in El Hierro; although it is not documented in Fuerteventura, it may have been present previously. According to Don Severo, a puyón fisherman, he and his sons caught up to 80 kg of parrotfish each day by swimming with the puyón. Information about this technique can be seen in the Corporación de Radio y Televisión Española (RTVE) report as part of the broadcast ‘Reduce Your Footprint’ and is available at this link (https://www.youtube.com/watch?v=Vaz2p6W1BCI).

Although a hook-and-line fishing pattern is observed, no artifacts identifiable as such have been found in the archaeological record of Fuerteventura. However, the lack of specialized studies on the bone and mollusk industries on the island currently prevents ruling out the presence of this type of tool, which includes crow hooks, bipointed, composite, and others. Additionally, since wood or plant fibers are not preserved, we cannot rule out that some of these implements were made from plant material. It should also be noted that these hooks are known on other islands and that in Gran Canaria they are made from pig’s tusks (e.g. Rodríguez Santana 1996; Santana et al. 2026).

Proposing fishing activities for the other identified species is challenging due to the scarcity of osteometric information, which limits the ability to accurately estimate the techniques and methods used. However, strictly focusing on the documented taxa, the use of hook-and-line gear seems evident. Still, the capture of fish in intertidal pools cannot be ruled out, as specimens susceptible to capture using techniques such as cast netting, are recorded, including the salema (*S. salpa*) and, in the case of sexually immature individuals, the dusky grouper (*E. marginatus*). This possibility is also supported by written sources that mention the use of artificial intertidal pools in conjunction with the poison fish technique (Rodríguez Santana 1996).

The record also includes species we could call ‘deep coastal habitat species,’ that is, fish associated with natural reefs or open-ocean spaces characterized by flat and steep beaches, mixed bottoms, caves, and rocky crevices. These habitats, especially abundant on the northwest and west coasts of Fuerteventura, host species such as the white damselfish (*Chromis limbata*, Valenciennes, 1833), the trumpetfish (*Aulostomus strigosus*, Wheeler, 1955), and moray eels (Muraenidae), all of which are associated with caves and crevices in open areas.

**Fig. 7:**
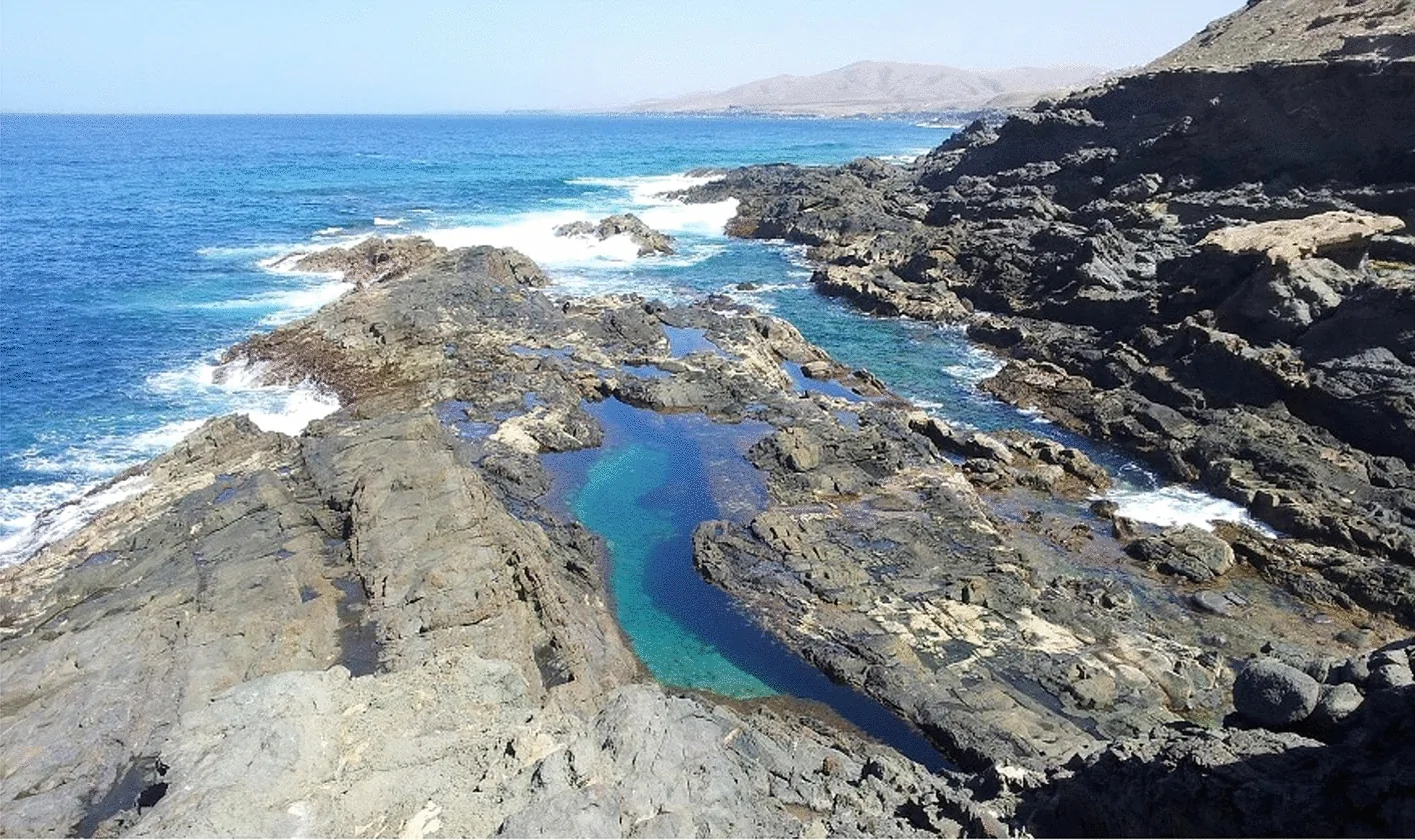
Example of a set of tide pools at El Valle beach, on the western coast of Fuerteventura. On the right, intertidal pools with notable depths during low tide are identified, while on the left, fishing areas are observed that maintain a significant depth even at low tide, reflecting the diversity of coastal habitats exploited for fishing

The presence of these species also suggests the possible use of fish traps or trammel nets fixed on steep rocky promontories, called “mangas,” which extend into the sea and allow access to great depths (Fig. 7). These structures, located on flat beaches with mixed bottoms and caves, create small patches of heterogeneous habitats, making them ideal spots for fishing. The concentration of these conditions in certain areas of the island explains the abundance of deep habitat species in those sectors, indicating a selective exploitation of the environment based on the topography and the diversity of coastal microhabitats.

The size and weight estimates for various species also highlight the Majo’s ability to catch relatively large and heavy animals. Notably, they have captured a shark and a dusky grouper measuring around one meter in length, with estimated weights of 5 kg and 17 kg, respectively. Additionally, there are also zebra seabream, island groupers, and redbranded seabream, which measure approximately 80 cm and weigh between 4 and 8 kg.

A very important element revealed by the study of these three sites is the presence of marine animal remains both on the coast and inland on the island of Fuerteventura. It is worth noting that at Llano del Sombrero, the collection of material was not done with fine mesh, which certainly partly explains the very low number of fish remains. Considering the overall archaeozoological information and the nature of each site (residential, functional), it is possible to propose a model of marine exploitation related to the dynamics of land occupation. This model is not exclusive to Fuerteventura, but corresponds to an exploitation system also observed throughout the rest of the archipelago. Thus, it is possible to identify the presence of sites specialized in fish processing. In Fuerteventura, the sites of Punta del Mallorquín (this study) and Cueva del Junquillo (Suleiman Ruiz and López Guerrero 2025) are presented as two typical sites for immediate processing of marine resource exploitation. In this way, once processed (possibly smoked or sun-dried) in these spaces, it is thought that the fish, along with some bones and perhaps the last caught fish, were transported to the habitation areas. This model would then imply a decrease in the number of fish remains in the residential spaces, but possibly not in the amount of fish meat consumed by the inhabitants, at least in Fuerteventura.

Regarding the processing of the fish, the multidisciplinary study conducted at the Playa Chica site in Gran Canaria, provides evidence of the use of smoking as a fish preservation technique (Santana et al. 2026). The same technique may have been used in Fuerteventura, but more studies are needed to confirm this. Ethnographic records in Fuerteventura also document the practice of “jarear” (salting and drying) the fish in the sun after removing the guts (Monesma 2021). This practice is still in use among the older population, which could also explain the higher presence of parrotfish fish bones in the archaeological sites. In fact, with this practice, nearly all of the animal’s bones remain in their anatomical position until consumption. The presence of complete or nearly complete parrotfish fish neurocrania in the residential area of the Villaverde Cave, along with the few cut marks, seems to support the existence of this practice.

Regarding the processing and transportation system of the fish, the chi-square statistical test conducted on the observed frequency difference between the number of parrotfish (*p* = 0.0001) from Cueva de Villaverde and Punta del Mallorquín, as well as the Fisher’s exact test (*p*-value much less than 0.05) between the number of moray eels recorded at these two sites, demonstrates the existence of a statistically significant difference. This difference could be explained precisely by a different processing and transportation system for the various fish species. In this way, the parrotfish would be transported whole, while moray eels (and perhaps other fish) would be processed near the fishing site, such as Punta del Mallorquín, before being transported. The high frequency of parrotfish in Cueva de Villaverde (93%) seems to support this hypothesis of a privileged accumulation of this species in an area not dedicated to processing marine resources. Additionally, the percentage of skeletal part representation (%SPR) shows that the bones of the neurocranium and viscocranium of the parrotfish are much more represented in Cueva de Villaverde (dentale: 21% and basioccipitale: 8%) than in Punta del Mallorquín (dentale: 3% and no basioccipitale). Conversely, the %SPR of pharyngeal bones indicates a slightly higher representation in Punta del Mallorquín (76% vs. 60%) than in Cueva de Villaverde. This may be related to the fact that the pharyngeal bones can be removed during fish cleaning. However, this difference could also reflect environmental variation and the presence of different fish species compositions in the explored areas. This hypothesis should be tested in future studies as new, well-contextualized ichthyofaunal records are analyzed.

Finally, it is interesting to note the difference in the percentage of charred bones between Punta del Mallorquín (23%) and Cueva de Villaverde (5%). This difference also seems to support the variation in activities between the two sites, with greater processing and preparation of fish at Punta del Mallorquín.

## Conclusion

The data obtained demonstrate the importance of the sea for the Majos throughout all phases of the island’s occupation, with ichthyoarchaeological records from the initial phase until the conquest by Europeans. This importance of the sea is also reflected in the practice of shellfish gathering and in the possible exploitation of marine animals such as turtles and the monk seal (*Monachus monachus*) (Brito-Mayor et al., under review). That study also suggests that the role of the sea for the Majos cannot be understood solely by observing the number of fish bones within a particular site, but is integrated into a dynamic system of exploration and occupation of the territory, traversed by both goods and humans.

The evidence presented allows us to place Fuerteventura within an island archaeology that recognizes marine or coastal landscapes alongside other spaces dedicated to agricultural and pastoral economies as central axes of economic and symbolic organization, rather than as mere appendages of pastoral and agricultural economies. The record of fish diversity, the proportion of maritime catch, and the consistent pattern of capturing adult-sized fish all indicate long-standing, specialized fishing techniques used over centuries. Coupled with evidence of fish processing and the links between coastal processing sites and domestic settlements, this data challenges traditional narratives that are overly focused on terrestrial activities. Instead, it suggests a more complex model where the sea influences logistics, seasonal patterns, and supply networks. These strategies highlight the role of marine exploitation in insular colonization, the shaping of coastal landscapes, and technological specialization as drivers of resilience during Fuerteventura’s indigenous period.

In addition to its interpretive scope, the work opens a relatively underdeveloped research field in the Canary Islands: ichioarchaeology as a direct means to quantify trophic contributions, reconstruct fishing techniques, and model mobility circuits between processing sites along the coast and settlements. By demonstrating that counts of fish remains (NISP/MNI) can overestimate the dominance of certain species compared to less abundant but higher biomass species, and by revealing processing and distribution chains that distort archaeological representation, the study provides methodological tools to reevaluate indigenous diet and economy beyond traditional indicators. This study strengthens a comparative agenda for the archipelago: expanding systematic sampling with fine meshes and/or flotation, delving into ichthyometric and taphonomic analysis, and combining ichthyoarchaeology series to refine dietary estimates and test hypotheses about economic strategies during the indigenous period.

## Supplementary Information

Below is the link to the electronic supplementary material.

Supplementary file1 (XLSX 2252 KB)

## Data Availability

No datasets were generated or analysed during the current study.
